# Optimal number of atlases and label fusion for automatic multi-atlas-based brachial plexus contouring in radiotherapy treatment planning

**DOI:** 10.1186/s13014-015-0579-1

**Published:** 2016-01-07

**Authors:** Joris Van de Velde, Johan Wouters, Tom Vercauteren, Werner De Gersem, Eric Achten, Wilfried De Neve, Tom Van Hoof

**Affiliations:** Department of Anatomy, Ghent University, De Pintelaan 185, 9000 Ghent, Belgium; Department of Radiotherapy, Ghent University, De Pintelaan 185, 9000 Ghent, Belgium; Department of Radiology, Ghent University, De Pintelaan 185, 9000 Ghent, Belgium

**Keywords:** Automatic, Multi-atlas-based, Brachial plexus, Segmentation, Label fusion, Cadavers

## Abstract

**Background:**

The present study aimed to define the optimal number of atlases for automatic multi-atlas-based brachial plexus (BP) segmentation and to compare Simultaneous Truth and Performance Level Estimation (STAPLE) label fusion with Patch label fusion using the ADMIRE® software. The accuracy of the autosegmentations was measured by comparing all of the generated autosegmentations with the anatomically validated gold standard segmentations that were developed using cadavers.

**Materials and methods:**

Twelve cadaver computed tomography (CT) atlases were used for automatic multi-atlas-based segmentation. To determine the optimal number of atlases, one atlas was selected as a patient and the 11 remaining atlases were registered onto this patient using a deformable image registration algorithm. Next, label fusion was performed by using every possible combination of 2 to 11 atlases, once using STAPLE and once using Patch. This procedure was repeated for every atlas as a patient.

The similarity of the generated automatic BP segmentations and the gold standard segmentation was measured by calculating the average Dice similarity (DSC), Jaccard (JI) and True positive rate (TPR) for each number of atlases. These similarity indices were compared for the different number of atlases using an equivalence trial and for the two label fusion groups using an independent sample-*t* test.

**Results:**

DSC’s and JI’s were highest when using nine atlases with both STAPLE (average DSC = 0,532; JI = 0,369) and Patch (average DSC = 0,530; JI = 0,370). When comparing both label fusion algorithms using 9 atlases for both, DSC and JI values were not significantly different. However, significantly higher TPR values were achieved in favour of STAPLE (*p* < 0,001). When fewer than four atlases were used, STAPLE produced significantly lower DSC, JI and TPR values than did Patch (*p* = 0,0048).

**Conclusions:**

Using 9 atlases with STAPLE label fusion resulted in the most accurate BP autosegmentations (average DSC = 0,532; JI = 0,369 and TPR = 0,760). Only when using fewer than four atlases did the Patch label fusion results in a significantly more accurate autosegmentation than STAPLE.

## Background

Manual brachial plexus (BP) segmentation on planning computed tomography (CT) for radiation therapy treatment planning is a time-consuming and unreliable process [1]. An effective automatic BP contouring method could relieve clinicians of this tedious task and would result in a higher inter- and intra-observer reliability and accuracy of the contouring process [2]. This issue is of growing importance following the introduction of both function-sparing and adaptive intensity modulated radiation therapy (IMRT), where the number and frequency of delineation of organs at risk (OAR’s) are increased [3, 4].

The BP is one of the OAR’s in IMRT for head-and-neck, lung and breast cancer patients. However, in clinical practice, the BP is often not delineated during treatment planning, and when the delineation is included, the accuracy tends to be low [1]. Incorrect or absent delineations of OAR’s in IMRT treatment planning however, have proven to be a main source of uncertainty in historical dose-volume effect data, which leads to the reduced performance of predictive models [5]. Moreover, when the BP radiation dose is not controlled during IMRT treatment, the possibility exists that the dose to this organ exceeds the BP tolerance dose of 66 Gy [6–8], which can potentially cause radiation-induced brachial plexopathy (RIBP). RIBP was thought to be uncommon for head-and-neck cancer patients, but recent clinical investigations have suggested that it remains underreported [9, 10].

To develop accurate automatic multi-atlas-based BP segmentations, multiple parameters must be controlled.

The first parameter is the optimal image registration and label fusion algorithm that are used. In multi-atlas-based autosegmentation strategies, several available presegmented images –called atlases– are first registered separately to the patient using deformable image registration. During the deformable image registration process a deformation vector field (DVF), describing the non-linear transformation from a presegmented image dataset to a patient image dataset, is created. Based on the computed DVF a set of delineations on the presegmented image data set are deformed on the patient image data set. The series of deformed delineations on the patient image data set are combined by the label fusion algorithm to obtain a unique and final consensus segmentation. Multiple image registration and label fusion algorithms for various organs have been compared in the literature [11–14]. However, only one publication [15] investigated BP autosegmentation. In that study, the authors concluded that multi-atlas autosegmentation can be effectively used to delineate BP on CT. However, these conclusions may be unreliable because the autosegmentation itself and also the subsequent validation procedure were based on BP gold standards that were not validated [1]. Moreover, Yang et al. [15] used the Simultaneous Truth and Performance Level Estimation (STAPLE) algorithm to generate automatic BP contours. A described weakness of the STAPLE label fusion method is that it ignores the image data and uses only the segmentations when computing the label fusion [16]. In an attempt to counter this weakness, an additional intensity weighted label fusion method called ‘Patch’ fusion [17] was recently implemented in the ADMIRE® software. The ADMIRE® white paper reported that this Patch fusion outperforms the STAPLE algorithm for some anatomical structures with a very stable anatomical topography [18]. No publication has been found, however, in which the use of this label fusion algorithm was investigated for automatic multi-atlas-based BP segmentation.

A second parameter that must be controlled to obtain the maximum accuracy is the number of atlases that has to be used for multi-atlas-based BP autosegmentation. In different publications, multi-atlas-based automatic segmentation methods have proven to be more effective than single-atlas-based methods [19, 20] but the specific number of atlases to use was investigated in only a few publications [14, 21]. None of these studies provided a specific number for optimal automatic BP contouring.

The purpose of this study was to define the optimal number of atlases to use for automatic multi-atlas-based BP contouring and to compare the STAPLE algorithm with Patch label fusion using the ADMIRE® software. This was measured by comparing all of the generated automatic BP segmentations with high-quality, anatomically validated, gold standard atlases that were developed using cadavers.

## Materials and methods

To develop gold standard atlases for BP contouring, 12 cadavers (age and gender randomized) were used. The cadavers were embalmed according to Thiel because of their optimal image quality and movement capacities [22, 23]. The latter allowed for the required standardization of the scan position. Magnetic resonance imaging (MRI) of the head-and-neck region was performed to generate high-quality BP delineations that were anatomically validated by dissection. These anatomically validated, MRI-based, BP delineations were then rigidly fused to the corresponding CT to obtain BP gold standard delineations that were applicable to the radiation therapy planning system. A detailed description was provided by Van de Velde et al. [24]. This study was approved by the ethics committee of University Hospital Ghent (reference number: B67020142069), and was in compliance with the Helsinki Declaration.

For image registration and label fusion, the ADMIRE® software 1.10.02 (Elekta AB, Stockholm, Sweden) was used. ADMIRE® performs the segmentation of a novel subject image (here called ‘patient’) by using multiple pre-segmented images, which are also known as ‘atlases’. The ‘General’ algorithm in ADMIRE® is used for the initial deformable image registration [18]. This image registration framework consists of three major steps: a linear registration and two non-linear registration steps. With each step the number of degrees of freedom increases, and is used to provide initialization for the next step.

For label fusion, 2 different algorithms in ADMIRE® are compared: the STAPLE label fusion [16] and Patch label fusion [17]. The STAPLE algorithm works with a statistical framework that simultaneously estimates the underlying ‘truth’ segmentation and the accuracy of each individual atlas [18]. It ignores the image data and uses only the segmentations when computing the label fusion. In contrast, the Patch algorithm considers the accuracy of the initial image registration by comparing the intensity similarity between the atlas and the patient after being aligned, to get better label fusion results. This process, is called ‘intensity weighting’.

### Procedure

The present study aimed to determine the optimal number of atlases and to compare the STAPLE with the Patch label fusion algorithm for multi-atlas-based BP contouring in ADMIRE® software.

For this purpose, a leave-one-out strategy was followed. One of the 12 available cadaver CT-datasets was selected as a patient and the remaining CT-datasets, which contained the anatomically validated BP segmentation, served as atlases. All of the atlases were first registered separately onto the patient using the ‘General’ registration algorithm in ADMIRE®. Next, the label fusion was performed, with both STAPLE and Patch, first using every possible combination of 2 atlases. Subsequently, label fusion was repeated with a gradually increasing number of atlases, until every possible combination of 11 atlases was reached. This process was reiterated for every atlas as a patient. It resulted in 24432 combinations over the different number of atlases. A Power analysis was executed (power π = 80) to calculate the minimum sample size required for a 90 % confidence interval.

Next, for every generated ‘label fused’ autosegmentation, 3 similarity indices with the gold standard contour were calculated to quantify the accuracy (Table 1):

**Table 1:** Average Dice similarity coefficient, Jaccard index and True positive rate per number of atlases

		STAPLE			Patch		
Number of atlases	Samples	DSC (SD)	JI (SD)	TPR (SD)	DSC (SD)	JI (SD)	TPR (SD)
2	660	0,247 (0,179)	0,154 (0,131)	0,188 (0,158)	0,400 (0,157)	0,262 (0,124)	0,416 (0,178)
3	660	0,397 (0,184)	0,265 (0,151)	0,373 (0,187)	0,454 (0,157)	0,307 (0,136)	0,439 (0,163)
4	3960	0,472 (0,171)	0,325 (0,147)	0,473 (0,184)	0,477 (0,161)	0,328 (0,141)	0,445 (0,165)
5	5544	0,482 (0,153)	0,331 (0,132)	0,534 (0,166)	0,465 (0,149)	0,316 (0,128)	0,435 (0,150)
6	5544	0,519 (0,138)	0,362 (0,128)	0,616 (0,155)	0,501 (0,146)	0,347 (0,133)	0,465 (0,150)
7	3960	0,514 (0,129)	0,356 (0,117)	0,658 (0,147)	0,492 (0,144)	0,339 (0,131)	0,446 (0,142)
8	1980	0,501 (0,120)	0,343 (0,106)	0,686 (0,143)	0,501 (0,140)	0,346 (0,127)	0,466 (0,140)
9	660	0,532 (0,102)^a^	0,369 (0,940)^a^	0,726 (0,127)	0,530 (0,117)^a^	0,370 (0,112)^a^	0,466 (0,125)
10	132	0,510 (0,100)	0,349 (0,900)	0,742 (0,127)	0,524 (0,124)	0,365 (0,116)	0,468 (0,121)
11	12	0,506 (0,940)	0,344 (0,840)	0,760 (0,126)^a^	0,530 (0,122)	0,370 (0,115)	0,471 (0,115)^a^

Abbreviations: *DSC* dice similarity coefficient; *JI* Jaccard index; *TPR* true positive rate

^a^Highest index values

First, Dice similarity coefficient (DSC) was calculated between these 2 segmentations. The DSC measures the spatial overlap between the gold standard A and the registered image B, and is defined as DSC(A,B) = 2(A∩B)/(A + B) where ∩ is the intersection volume. The DSC is situated between 0 and 1, with 0 indicating no agreement and 1 indicating perfect agreement.

We also calculated the Jaccard index (JI) as the ratio of the intersection volume and the entire union volume of the delineations: JI(A,B) = (A∩B)/(AUB). The JI is also situated between 0 and 1, with 0 indicating no agreement and 1 indicating perfect agreement.

At last, True positive rate (TPR) was measured between the gold standard BP (A) and the registered BP (B). TPR is the intersection volume of these, divided by the gold standard BP: TPR = (A∩B/A). TPR is situated between 0 and 1 with 0 indicating no inclusion and 1 indicating the total inclusion of A by B.

Finally, for each number of atlases, average DSC, JI and TPR were calculated over the different combinations.

To determine the clinically relevant optimal number of atlases, an equivalence trial was conducted [25, 26]. An equivalence trial is used to demonstrate similarity between compared groups. It uses a confidence interval in which equivalence is claimed when the confidence interval of the difference in outcome between compared groups is within a predetermined equivalence margin. This equivalence margin represents a clinically acceptable range of differences. For this study, an equivalence margin of 10 % was predetermined.

Only DSC and JI were appropriate as a reference for the equivalence trial, because in those indices, the most accurate segmentation will be associated with the highest index values, since both indices consider a penalty for false positive delineation area. The TPR from its side was not adequate for the equivalence trial because the highest TPR value does not necessary imply the most accurate segmentation [27], since a false positive delineation area is not penalized in this index.

DSC was chosen for equivalence trial over JI because the DSC has a linear course with an increasing correctly delineated volume and JI has not. Thus, a 10 % (= equivalence margin) increase or decrement of DSC always correlates with the same amount of increase or decrement of the correctly delineated volume [27]. Using JI conversely, the amount of correctly delineated volume associated with an increase or decrease of 10 % JI value, will vary depending on the starting value of the JI, because this index has a non-linear course. For example, an increase in JI value from 0.8 to 0.9 will result in a larger increase in percentage of correctly delineated volume than an increase from 0.2 to 0.3 [27].

Starting from the number of atlases with the maximal DSC values (reference group), the number of atlases was first gradually increased by one. If, by increasing the number of atlases each time starting from the reference group, the decrease of DSC (90 % CI) felt within the equivalence margin of 10 %, the groups were considered to be equivalent. This procedure was performed for the two label fusion groups separately [26]. Only in case of equivalent DSC values combined with significantly higher TPR values, the autosegmentation result was considered to be more accurate, because in this case the equivalence of the DSC values indicates that the increase of the false positive delineation area, which is not penalized by TPR, was kept within bounds.

Next, the number of atlases was gradually decreased by one, starting from the reference group. If, by decreasing the number of atlases each time starting from the reference group, the decrement of the DSC values fell within the equivalence margin, the calculation time could be reduced by using a lower number of atlases without clinically relevant loss in accuracy.

Thereafter, the difference between STAPLE and Patch label fusion was determined using an independent sample *t*-test. Therefore, in the 2 label fusion groups, the similarity indices for their respective clinically relevant optimal number of atlases were compared.

## Results

**Fig. 1:**
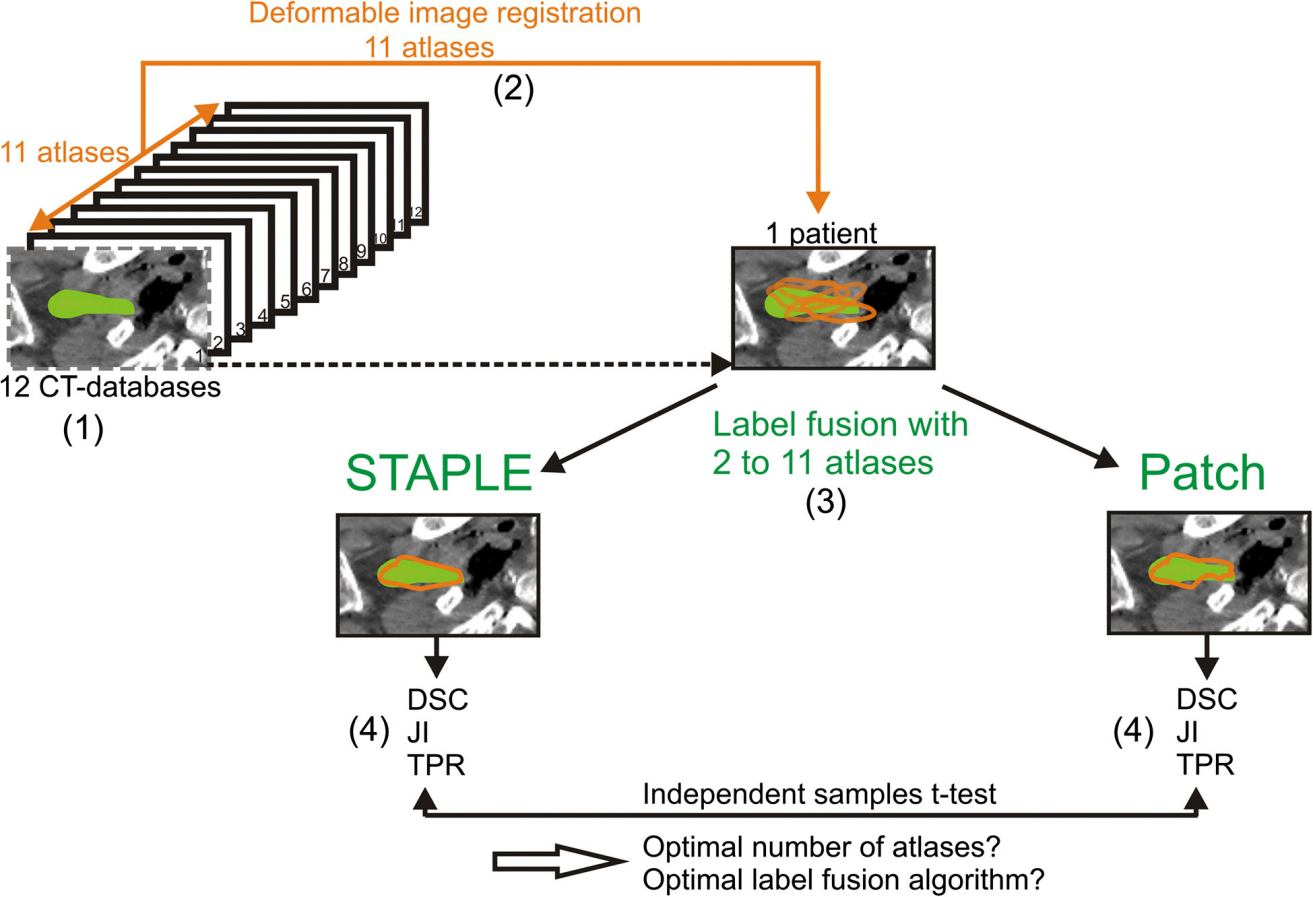
Schematic illustration of the procedure for determining the optimal number of atlases and optimal label fusion. (1) Twelve cadaver CT datasets were included, and one atlas was selected as a patient. (2) The 11 remaining atlases were used for deformable image registration on the patient. (3) Label fusion was performed with 2 up to 11 atlases, once using STAPLE and once using Patch. (4) For each number of atlases, the average Dice similarity coefficient (DSC), Jaccard index (JI) and True positive rate (TPR) were calculated for the generated contour (*orange*) with the gold standard contour (*green*). This procedure was repeated for every atlas as a patient

**Fig. 2:**
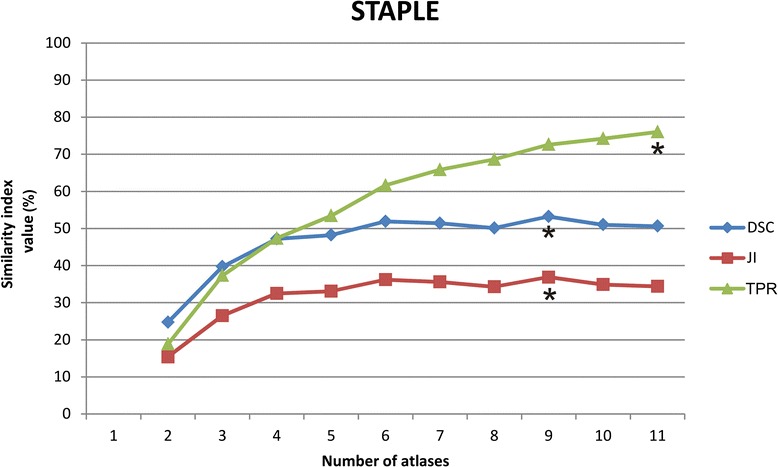
Behaviour of the average similarity indices with an increasing number of atlases fused using the STAPLE algorithm. * indicates the highest similarity index values

**Fig. 3:**
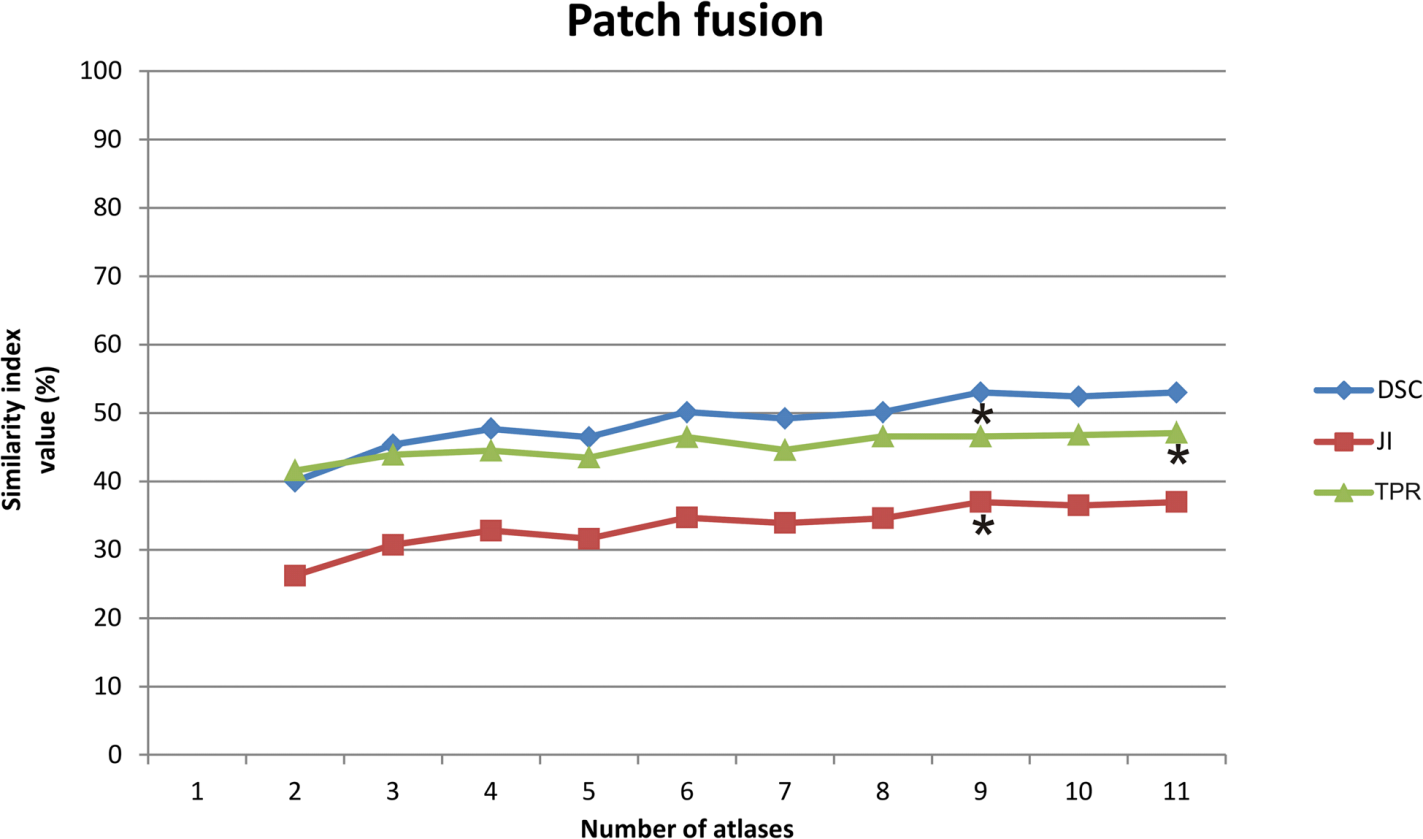
Behaviour of the average similarity indices with an increasing number of atlases fused using the Patch algorithm. * indicates the highest similarity index values

The power analysis (π = 80) resulted in a sample size of 150 combinations per number of atlases needed for a 90 % confidence interval. For each number of atlases, the average DSC, JI and TPR, their standard deviations and their possible combinations (samples) are shown in Table 1 and Figs. 1, 2 and 3 for both groups.

The highest average DSC and JI values were found when using 9 atlases for both STAPLE and Patch fusion (Table 1, Figs. 2 and 3). When more than 9 atlases were used, only TPR for STAPLE label fusion continued to increase (Fig. 2).

**Fig. 4:**
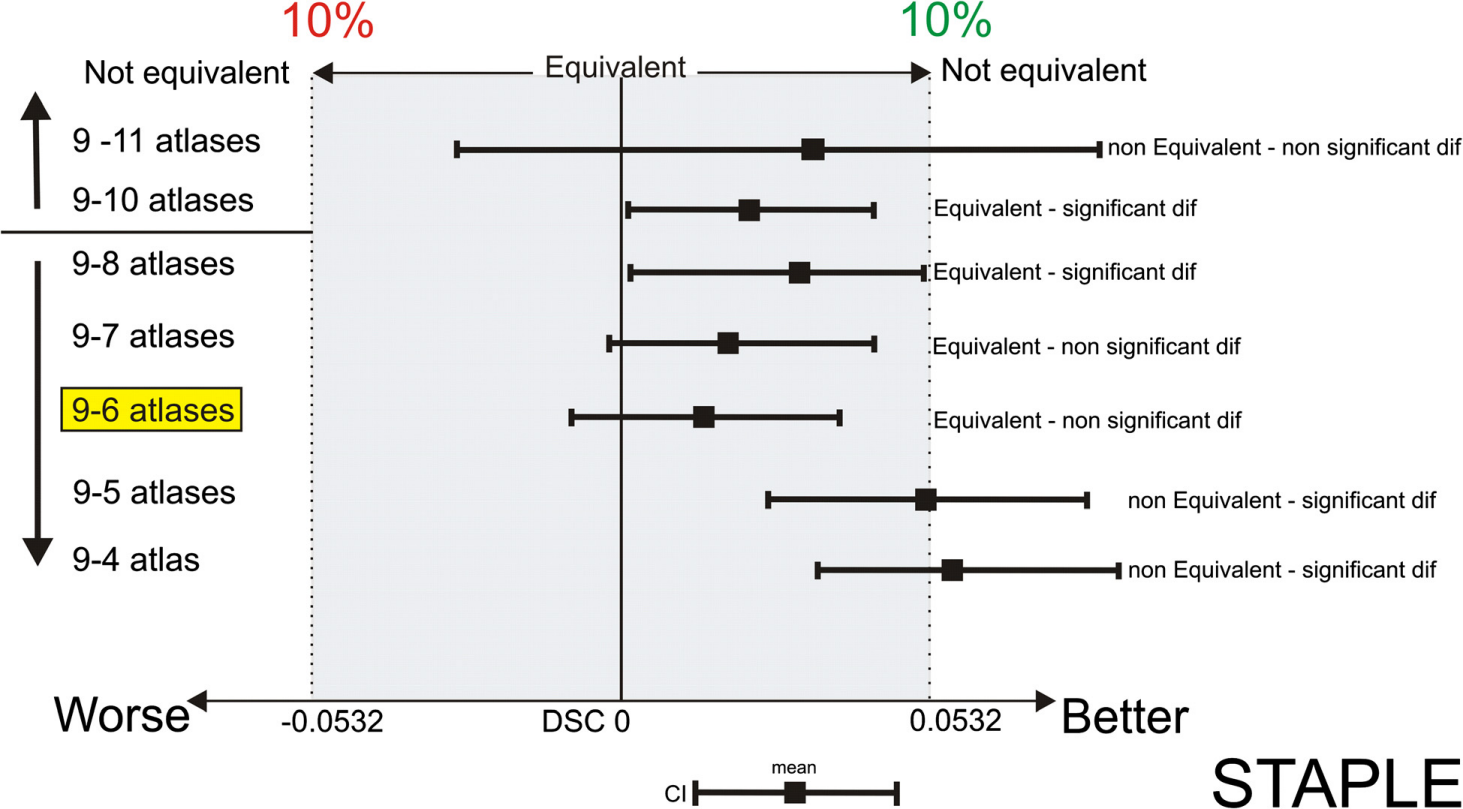
Interpretation of the equivalence using 9 atlases in multi-atlas brachial plexus autosegmentation compared to using more (10–11) and fewer atlases (8-7-6-5-4) with STAPLE label fusion. Using 6 atlases is equivalent to using 9 atlases (*yellow marked*). The shaded area covers the equivalence range of 10 %. □ = observed point estimate of the outcome difference in each number of atlases, corresponding error bar = two-sided 90 % confidence interval (caps at each end = lower and upper bar bounds of confidence interval). DSC, Dice similarity coefficient; CI, confidence interval

By increasing the number of atlases for STAPLE label fusion from 9 to 10 atlases, the decrease in DSC values still fell within the predisposed equivalence margin of 10 % (Fig. 4) but no significantly higher TPR values were achieved. When using 11 atlases, DSC were no longer equivalent to the results obtained when using 9 atlases. However, the number of possible combinations for a power of 90 (sample size of 150) was not sufficient with 10 and 11 atlases, so no definitive conclusions can be drawn concerning these number of atlases.

By decreasing the number of atlases from 9 to 6 atlases the decrease in DSC values still fell within the predisposed equivalence margin (Fig. 4). The average calculation time was reduced from 19 min to 17 min. When using fewer than 6 atlases, DSC values were no longer equivalent to the results obtained when using 9 atlases.

**Fig. 5:**
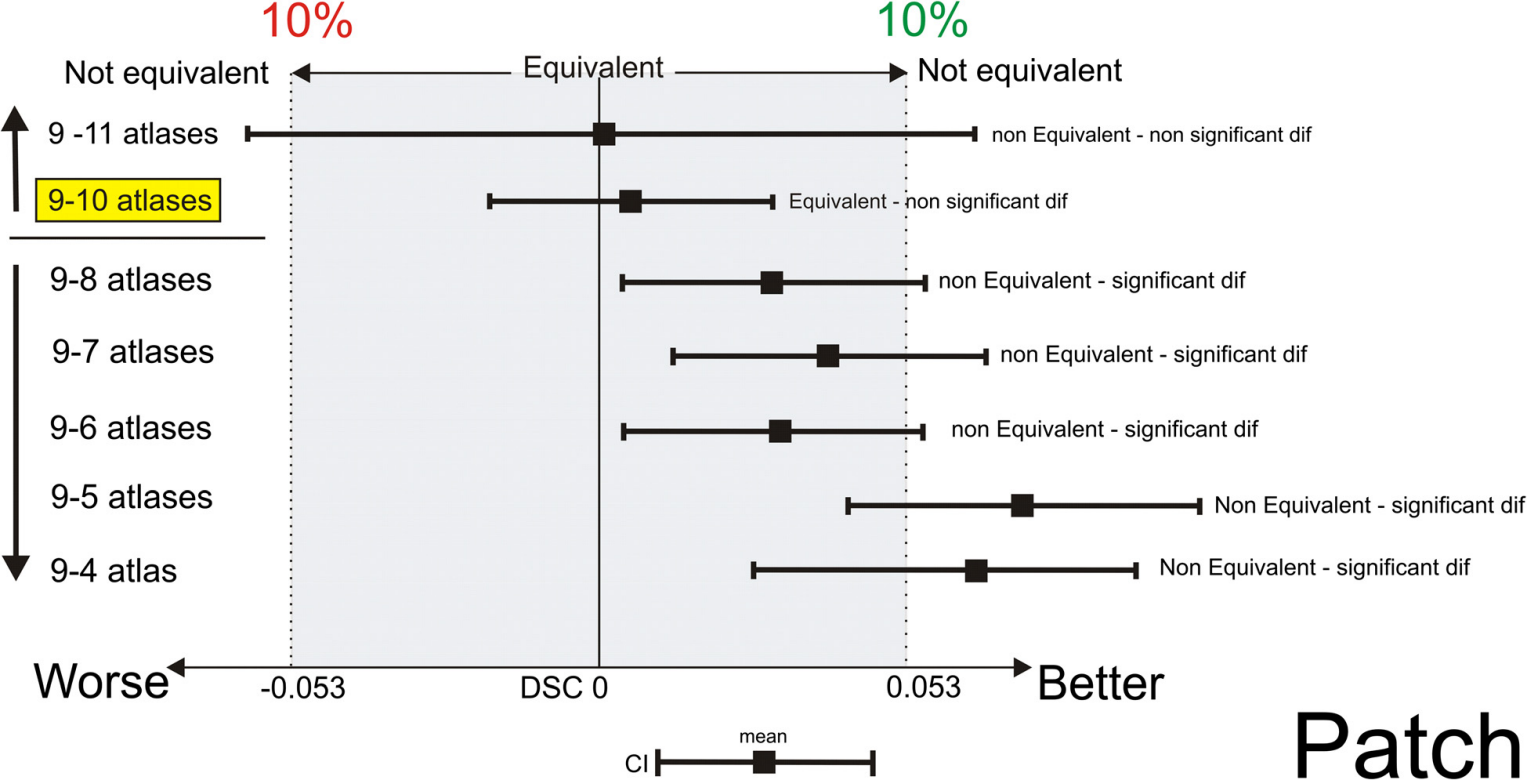
Interpretation of the equivalence using 9 atlases in multi-atlas brachial plexus autosegmentation compared with using more (10–11) and fewer (8-7-6-5-4) atlases with Patch label fusion. Using 10 atlases is equivalent to using 9 atlases (*yellow marked*). The shaded area covers the equivalence range of 10 %. □ = observed point estimate of the outcome difference in each number of atlases, corresponding error bar = two-sided 90 % confidence interval (caps at each end = lower and upper bar bounds of confidence interval). DSC, Dice similarity coefficient; CI, confidence interval

By increasing the number of atlases for Patch label fusion from 9 to 10 atlases, the decrease in the DSC values did fall within the predisposed equivalence margin of 10 % (Fig. 5) but no significantly higher TPR values were achieved. When the number of atlases was decreased until 8 or lower, the decrease in DSC was not within the equivalence margin (Fig. 5). Also here, the number of possible combinations for a power of 90 (sample size of 150) was not sufficient with 10 and 11 atlases.

When comparing the label fusion algorithms using the optimal number of atlases for both (9 for both STAPLE and Patch) DSC and JI values were not significantly different. However, significantly higher TPR values were achieved in favour of STAPLE (*p* < 0,001).

Only when fewer than four atlases were used, STAPLE resulted in significantly lower DSC, JI and TPR values than Patch (*p* = 0,0048).

## Discussion

The purpose of this study was to determine the optimal number of atlases for automatic multi-atlas-based BP contouring and to compare STAPLE and PATCH label fusion algorithms using the ADMIRE® software.

For STAPLE, the average DSC and JI values were maximal when using 9 atlases (Table 1). When the number of atlases was increased to 10, the DSC values remained equivalent to those obtained when using 9 atlases, but the TPR values were not significantly higher.

The most accurate autosegmentation results are achieved when the JI and DSC values reach their maximum value. The number of atlases where these maximum values are reached are identical for both similarity indices. From this point on, it is only possible to achieve higher TPR values by adding more atlases. However, when the TPR values increase and the DSC and JI values decrease, the increase in the true-positive delineation area is associated with a proportionally larger increase in the false positive delineation area. This may occur because TPR only measures the increase in the true-positive delineation area and does not penalize an increase of the false positive delineation area. DSC and JI, in contrast, do penalize an increase in the false-positive delineation area.

Because the decrease in DSC from 9 to 10 atlases fell within the equivalence margin, and because the TPR values were not significantly higher, autosegmentations obtained with 10 atlases could not improve the accuracy compared to those obtained using nine atlases for STAPLE. Consequently, the optimal number of atlases is 9 for STAPLE label fusion.

In the case of limited computer calculation power, six atlases could be used for STAPLE without a clinically relevant loss of accuracy and an average time saving of 2 min.

For Patch, the DSC and JI values were also maximal at nine atlases. By increasing the number of atlases to 10, no significant increase in TPR values was achieved either, which indicated that Patch fusion with nine atlases also resulted in the most accurate autosegmentations. To reduce the calculation time, the number of atlases cannot be decreased without a clinically relevant loss of autosegmentation accuracy (Fig. 5).

Comparing both label fusion algorithms (STAPLE and Patch) using their respective optimal number of atlases (9 for both), DSC and JI values were not significantly different. However, significantly higher TPR values were found in favour of STAPLE (*p* < 0,001). Therefore, we recommend using STAPLE label fusion with 9 atlases to obtain the most accurate autosegmentations results.

Conversely, when fewer than four atlases were used, STAPLE provided significantly less accurate results than did Patch (*p* = 0.004862). So, Patch label fusion is preferable over STAPLE when only less than 4 atlases are available.

The current study is the first to investigate the optimal number of atlases for BP autosegmentation. The optimal number of atlases for some other organs was already studied: for the nucleus caudatus Aljabar et al. [21] concluded that using eight atlases is optimal; for the hippocampus, the highest accuracy is reached with a selection of 25 atlases. Pirozzi et al. (2012) concluded that for the bladder and the femur, the optimal number of atlases was five, and that the optimal number for the prostate and rectum was four [14]. Remarkable is that in the first study [21], the number of atlases for autosegmentation of anatomically stable brain structures is higher than in the second study [14], in which anatomically variable organs were autosegmented. The opposite could be expected. The varying results of these studies only show that the optimal number of atlases is very organ-dependent and especially algorithm-dependent. So, for more general conclusions concerning the optimal number of atlases for BP autosegmentation, other algorithms also have to be investigated.

Few studies were found that compared ADMIRE® software to other autosegmentation software. Simmat et al. [11] found higher flexibility and robustness in the algorithm used in the ADMIRE® software compared with the algorithms in Iplan® [12] for the bladder, prostate and rectum. La Macchia et al. [13] found the best label fusion results using STAPLE in ADMIRE® compared with the algorithms in VelocityA® and MIM 5®, for the head-and-neck region. BP autosegmentation was not included in both studies. For general conclusions concerning the best autosegmentation software for BP autosegmentation, different autosegmentation software need to be compared in further studies.

Another limitation of the current study is that only 12 atlases were available. Hence, for the combinations with 10 and 11 atlases, the sample size was not big enough to draw definitive conclusions. To increase the statistical power and to draw definitive conclusions for the highest number of atlases, more atlases need to be included in the study. The more atlases included in the study, the more accurate the autosegmentation results will be as well, because the probability of selecting atlases that are more similar to the patient’s morphotype will increase.

The dosimetric implications of optimization of label fusion and the number of atlases on radiation therapy treatment planning were not included in the investigation. Additional studies are in process to study the dosimetric impact and measure the potential benefit for patients undergoing radiation therapy treatment. Future perspectives include further increasing the accuracy of the automatic BP segmentations to a clinically acceptable level, by combining the optimal number of atlases and label fusion with an effective atlas selection strategy and including higher number of anatomically validated atlases to study the effect of using more than 11 atlases.

## Conclusion

STAPLE is preferable to Patch label fusion for multi-atlas-based BP autosegmentation. Only when fewer than four atlases are available, it’s preferable to choose Patch above STAPLE.

Using nine atlases with STAPLE resulted in the most accurate BP autosegmentations. With a limited computer calculation power, the number of atlases could be decreased until 6 without a clinically relevant loss of accuracy.

## Abbreviations

BP: brachial plexus
STAPLE: simultaneous truth and performance level estimation
CT: computed tomography
DSC: dice similarity coefficient
JI: Jaccard index
TPR: true positive rate
IMRT: intensity modulated radiation therapy
OAR: organs at risk
MRI: magnetic resonance imaging
